# Longitudinal fecal cortisol metabolite concentrations across the transition period in multiparous dairy cows under commercial conditions

**DOI:** 10.1093/tas/txag174

**Published:** 2026-10-28

**Authors:** Cristina Castillo, Lucia Díaz, Elena Niceas Martínez, Rodrigo Muiño, Joaquín Hernández

**Affiliations:** Departamento de Patoloxía Animal, Facultad de Veterinaria de Lugo, Campus Terra-IBADER, Universidad de Santiago de Compostela, Lugo, 27002, Spain; Departamento de Patoloxía Animal, Facultad de Veterinaria de Lugo, Campus Terra-IBADER, Universidad de Santiago de Compostela, Lugo, 27002, Spain; Departamento de Patoloxía Animal, Facultad de Veterinaria de Lugo, Campus Terra-IBADER, Universidad de Santiago de Compostela, Lugo, 27002, Spain; Departamento de Patoloxía Animal, Facultad de Veterinaria de Lugo, Campus Terra-IBADER, Universidad de Santiago de Compostela, Lugo, 27002, Spain; Departamento de Patoloxía Animal, Facultad de Veterinaria de Lugo, Campus Terra-IBADER, Universidad de Santiago de Compostela, Lugo, 27002, Spain

**Keywords:** adrenocortical activity, dairy cows, fecal cortisol metabolites, HPA axis, low-disturbance sampling, transition period

## Abstract

This longitudinal observational field study characterized fecal cortisol metabolite (FCM) concentrations across the transition period in multiparous Holstein-Friesian dairy cows under commercial conditions. The dataset comprised 38 clinically healthy cows from four farms, with 19 sampled in winter and 19 different cows in summer. Each cow was sampled at 1 month and 1 week before parturition and 1 week and 1 month after parturition, yielding 152 observations. FCM concentrations were quantified using an 11-oxoetiocholanolone enzyme immunoassay and analyzed using a mixed-design repeated-measures general linear model, with stage as the within-cow factor and season and farm as between-subject factors. FCM concentrations differed across transition stages (*P* < 0.001); estimated marginal means (95% CI) were 421.1 (363.5–478.8), 342.2 (288.8–395.6), 238.5 (205.6–271.5), and 226.1 (171.1–281.0) ng/g dry feces, respectively. Neither season (*P* = 0.815) nor the Stage × Season interaction (*P* = 0.213) was significant. Significant farm-related effects and interactions indicated heterogeneity among the sampled herds without establishing causal effects of management practices. Temperature-humidity index values described environmental conditions only. These findings indicate an overall prepartum-to-postpartum decline, although not all pairwise comparisons were significant. Longitudinal FCM profiling may complement investigations of adrenocortical activity during the transition period. Further studies integrating endocrine, clinical, productive, behavioral, and environmental indicators are needed to establish its relevance to dairy cow health, productivity, and welfare.

## Graphical Abstract

**Graphical Abstract:**
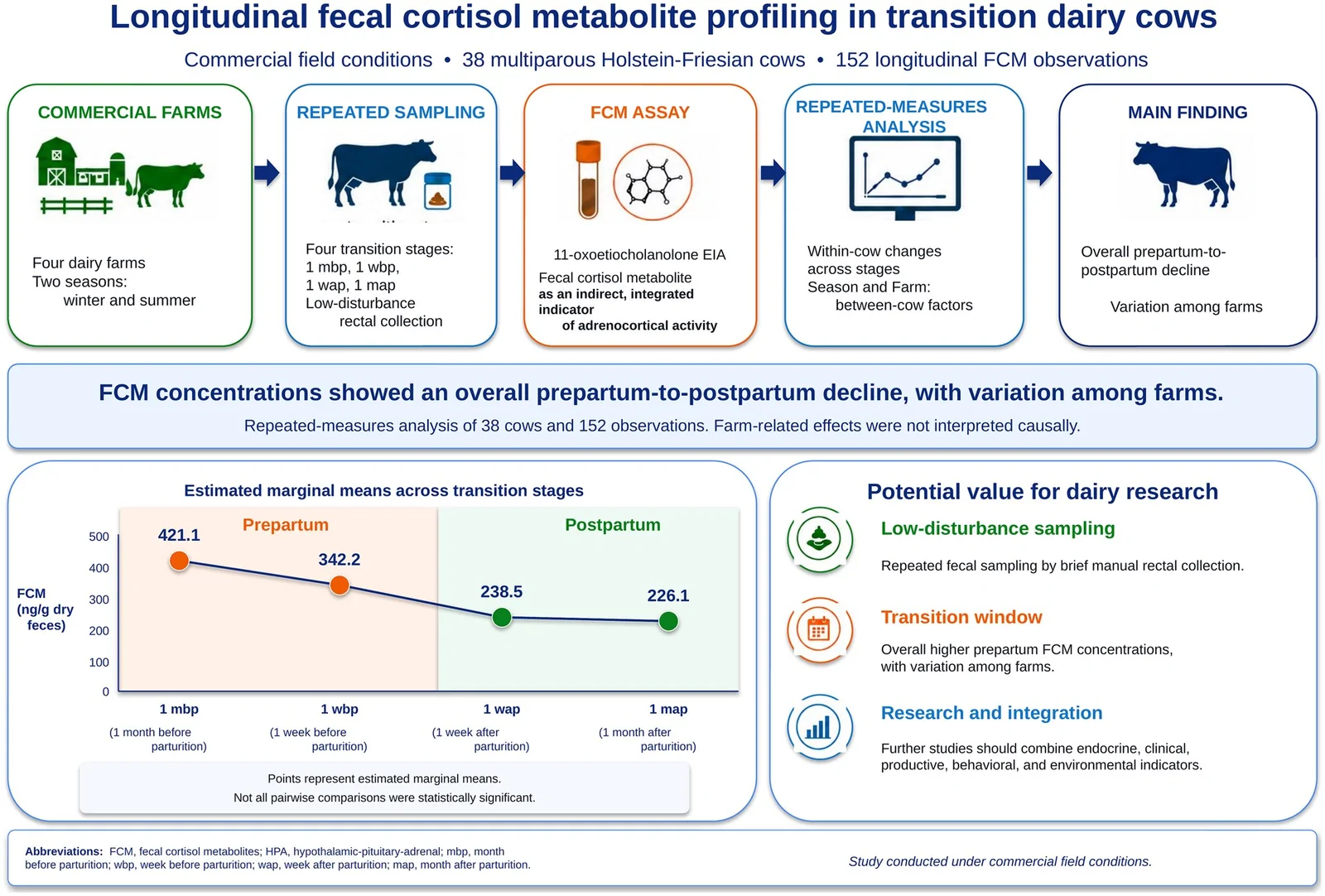

## Teaser

Repeated fecal cortisol metabolite profiling identified an overall pattern of higher prepartum than postpartum FCM concentrations in multiparous dairy cows under commercial conditions. This low-disturbance approach may complement future studies of adrenocortical activity during the transition period.

## Introduction

The transition period in dairy cows, defined as the interval from three weeks before to three weeks after calving, is the phase of greatest physiological and metabolic challenge in the productive life of the cow. During this period, animals undergo profound nutritional and metabolic adaptations associated with parturition, colostrum synthesis, and the onset of lactation. These changes are compounded by management practices and environmental conditions that can act as additional stressors. Consequently, transition management has major implications for health, welfare, subsequent lactation performance, and reproductive success (Abuelo 2025; Salamone et al. 2026). Animal welfare considerations in dairy production systems have also emphasized the importance of monitoring physiological stress responses under commercial conditions (EFSA 2015; EFSA AHAW 2023).

Oxidative stress, defined as a biochemical imbalance between reactive oxygen species and antioxidant defenses, is commonly used to describe some of the endocrine and metabolic shifts occurring during this phase. However, oxidative stress differs from adaptive stress, which reflects the hypothalamic-pituitary-adrenal (HPA) response to a stressor and supports the restoration of physiological homeostasis (Tsigos et al. 2020). Adaptive stress reflects the coordinated activation of neuroendocrine pathways that enable animals to cope with environmental and physiological challenges (Herman et al. 2016; de Bruijn and Romero 2018; Tsigos et al. 2020).

In this context, fecal cortisol metabolites provide an integrated measure of adrenocortical activity and may contribute to the investigation of physiological stress responses in dairy cows, as they reflect sustained HPA-axis activity associated with management or environmental challenges (Ebinghaus et al. 2020).

Compared with serum cortisol measurements, which require animal handling and may themselves induce stress, fecal sampling provides a less invasive estimate of endogenous cortisol secretion. FCM concentrations are less affected by acute fluctuations or pulsatile cortisol release and instead capture medium-term activation of the HPA-axis in response to adverse conditions (Idris et al. 2024).

Baseline FCM concentrations in adult cattle typically range from 10 to 185 ng/g of dry feces, with lower values observed in healthy, unstressed animals and higher values reported during late lactation or under stressful management conditions (Bertulat et al. 2013; Ebinghaus et al. 2020). Preliminary unpublished observations in multiparous Holstein-Friesian dairy cows suggested considerable inter-individual and between-farm variability in FCM concentrations during pregnancy. These observations supported the need for a longitudinal field study focused primarily on FCM dynamics during the transition period.

Evidence describing FCM responses during specific physiological stages, particularly the dairy cow transition period, remains limited under commercial farming conditions. Field studies also include unavoidable variation related to season, thermal context, management routines, and barn structure. Such variation can be useful for describing the real commercial context in which biomarkers are measured, but farm-level descriptors require careful interpretation when they are not independently replicated at the farm level.

Despite increasing interest in non-invasive stress biomarkers, the practical applicability of fecal cortisol metabolite profiling under commercial dairy conditions remains insufficiently validated. In particular, longitudinal evidence capturing within-animal variation across the transition period is scarce, and the relative importance of physiological stage compared to environmental or management-related contextual factors is not well established. Addressing this gap is important to determine whether FCM measurements may contribute to applied dairy herd monitoring under commercial conditions.

We hypothesized that FCM concentrations would vary across transition stages, with an overall pattern of higher prepartum than postpartum concentrations, consistent with variation in adrenocortical activity across the transition period. Therefore, the primary objective of this longitudinal observational field study was to characterize within-cow changes in FCM concentrations across the transition period in multiparous dairy cows under commercial conditions. A secondary objective was to document the commercial field conditions under which FCM measurements were obtained, without attempting to test farm-level management, production-system, or structural effects.

## Materials and methods

### Animal care and ethics statement

Ethical review and approval were waived because all animal procedures were low-disturbance, were performed by licensed veterinarians as part of routine clinical practice, and constituted non-experimental veterinary work. The Bioethics Committee of the University of Santiago de Compostela (Spain) confirmed that no formal ethical approval or project registration was required under current legislation.

### Environmental conditions

Environmental conditions, including temperature, humidity, and housing characteristics, are known to influence heat load and physiological stress responses in dairy cattle (Herbut et al. 2018; Oliveira et al. 2025).

As a general indicator of thermal load across seasons, the temperature-humidity index (THI) was calculated using mean daily ambient temperature and relative humidity recorded both inside and outside the barns, applying standard equations (Mader et al. 2006). In the present study, THI was included only as a contextual descriptor of the thermal environment, following the approach used in a previous experiment (Martínez et al. 2025). The objective was not to analyze seasonal THI differences but to contextualize the environmental conditions under which FCM concentrations were assessed during the transition period.

Temperature and humidity data were recorded using a Multi-Use Compact PDF Temperature and Humidity USB Data Logger (Multicomp Pro, Farnell Inc., U.K.), installed on each of the four farms at a height of 2 m within the lactation area. The device measures temperatures from −30°C to 70°C with an accuracy of ±0.5°C and relative humidity from 0% to 100% with an accuracy of ±3%. Measurements were recorded at hourly intervals and subsequently used to calculate THI according to the following formula (Mader et al. 2006):


THI=(0.81×T)+[(RH/100)×(T-14.4)]+46.4

where THI is the temperature-humidity index, T is air temperature (°C), and RH is relative humidity (%). In this equation, the coefficient 0.81 places greater weight on air temperature, reflecting its primary contribution to heat stress, whereas the term (T – 14.4) represents deviation from the thermal comfort threshold (14.4°C) and is modulated by relative humidity. The constant 46.4 calibrates the index based on empirical validation. THI values were used only to characterize the environmental context of the study.

The temperature-humidity index was not used to diagnose thermal stress or to assess short-term heat-load dynamics. The absence of continuous microclimatic variables, such as diurnal THI variability, radiant heat load, or airflow at animal level, precludes direct attribution of stress responses to thermal stress. Because THI was summarized as a mean farm-season value, the present analysis did not characterize maximum THI values or the duration of exposure above specific heat-load thresholds, which represents an additional limitation when interpreting thermal conditions.

### Animals, farms, and longitudinal field study design

This longitudinal observational field study was conducted on four commercial dairy farms. The main analytical focus was the repeated assessment of FCM concentrations across four transition stages within cows. Farm-level information was recorded to describe the commercial setting and to contextualize the variability inherent to field conditions.

Four commercial dairy farms were included and are referred to as Farms 1–4. Farm identity was included as a fixed between-subject factor to account for heterogeneity among the four sampled herds. Farm-level management and structural descriptors were recorded only to characterize the commercial context. Because these characteristics were not independently replicated across farms, they were not evaluated as causal predictors.

Multiparous transition cows were systematically selected from the four commercial farms during winter and summer. The analyzed cohort comprised 38 multiparous Holstein-Friesian cows, with 19 cows sampled during winter and 19 during summer. The distribution across farms was 10 cows from Farm 1, 8 from Farm 2, 10 from Farm 3, and 10 from Farm 4. Winter and summer groups consisted of different cows from the same farms; therefore, Season represented a between-cow factor.

Although the classical transition period is often defined as the interval from approximately 3 weeks before to 3 weeks after calving, the present sampling schedule was designed to characterize a broader longitudinal trajectory surrounding this period. Each cow was sampled at four stages: 1 month before parturition, 1 week before parturition, 1 week after parturition, and 1 month after parturition. The repeated-measures analysis therefore included 38 cows with four FCM measurements per cow, yielding a total of 152 longitudinal FCM observations. Selection was based on clinical examination, including body condition score (3.0–3.5), rectal temperature, thoracic and abdominal health, and mammary gland status, following established guidelines (Nelson et al. 2022). The study periods were defined as winter (WIN; January-April) and summer (SUM; June-September), corresponding to a total sampling duration of eight months. Mean ambient temperatures ranged from 0 to 15°C during WIN and from 15 to 30°C during SUM.

All cows were fed a basal total mixed ration (TMR) composed of corn silage, grass silage, and a formulated concentrate containing corn, rapeseed meal, soybean meal, barley, and beet molasses. Diets were formulated to meet the nutritional requirements of prepartum and early-lactation cows and were kept comparable across the four geographically proximate farms to minimize nutritional confounding. However, individual dry matter intake, individual energy balance, and cow-level feed availability were not measured; therefore, these variables could not be included in the statistical models and were not evaluated as experimental factors. The prepartum and postpartum feeding regimens were maintained consistently during their respective study periods and were unchanged between the winter and summer sampling periods. Thus, the diets summarized in Table 1 represent the stable prepartum and postpartum feeding regimens used in both seasons. The dietary composition presented in Table 1 was supplied by Aira SCG (Galicia, Spain), which formulated the diets using NDS Pro software (version 6.5, RUM&N SAS RE, Italy), based on the Cornell Net Carbohydrate and Protein System (CNCPS). Component-specific information distinguishing formulation estimates from laboratory-verified values was not provided to the authors.

**Table 1:** Diet characteristics common to all four farms and maintained across both study seasons.

Item	Before parturition (BP)	After parturition (AP)
**DM, g/kg FM**	685.3	481
**CF, g/kg DM**	253.2	172.8
**aNDFmo, g/kg DM**	480	324.4
**ADF, g/kg DM**	308.2	209.6
**ADL, g/kg DM**	47.5	32.6
**CP, g/kg DM**	143.6	157
**Starch, g/kg DM**	66	232
**EE, g/kg DM**	27.8	35.3
**Ashes, g/kg DM**	91.4	76.8

Abbreviations: DM, dry matter; CF, crude fiber; aNDFmo, neutral detergent fiber with heat-stable amylase and residual ash; ADF, acid detergent fiber; ADL, acid detergent lignin; CP, crude protein; EE, ether extract. Dry matter (DM) content is expressed as g/kg fresh matter. All other components are expressed as g/kg DM. Composition values were supplied by the diet formulation company; component-specific laboratory verification was not documented to the authors.

### Sample handling and laboratory analysis

Approximately 200 g of fecal material was obtained by brief manual rectal collection using a gloved hand between 09:00 and 11:00 h. This fixed sampling window was used throughout the study to standardize the collection protocol and reduce temporal variability among repeated measurements, including potential variation associated with time of day. Samples were placed in airtight plastic containers, labeled, immediately stored on ice, and transported under cooled conditions to the laboratory of Research Group No. 920694 (Endocrinología, Reproducción y Oncología Animal) at the Complutense University of Madrid (https://www.ucm.es/grupos/grupo/100). Upon arrival, samples were frozen at -20°C and maintained at this temperature until processing. This procedure ensured prompt refrigeration and freezing, minimizing the risk of FCM degradation.

Each fecal sample was processed individually. Samples were dried at 100°C for 2 h and subsequently pulverized. Then, 0.5 g of fecal powder was weighed into a 7 mL plastic tube, and 5 mL of 80% methanol was added. Tubes were shaken for 1 h and centrifuged at 500 × g for 15 min. The resulting supernatant, containing cortisol metabolites, was collected and stored in cryovials at −20°C until hormone analysis.

Fecal cortisol metabolite concentrations were determined using a group-specific enzyme immunoassay (11-oxoetiocholanolone EIA), as previously described (Möstl et al. 2002). This assay detects cortisol-derived metabolites rather than intact cortisol, which is extensively metabolized prior to excretion. The antibody used recognizes metabolites with similar structural characteristics (e.g., 11,17‑dioxoandrostanes), allowing sensitive and specific assessment of adrenocortical activity.

The assay is based on competitive binding, whereby fecal metabolites compete with an enzyme-labeled conjugate for antibody-binding sites. After incubation and washing, a chromogenic substrate was added to produce a colorimetric reaction. Absorbance was measured at 450 nm using a spectrophotometer and was inversely proportional to metabolite concentration.

Quantification was performed using a standard curve generated from known concentrations. Results were expressed as nanograms of fecal cortisol metabolites per gram of dry feces (ng/g). The assay detection range was 6.6–4000 pg/mL, with a 50 pg/mL extract included as a control. Intra-assay and inter-assay coefficients of variation were 2.8% and 4.0%, respectively.

### Statistical analysis

Statistical analyses were performed using IBM SPSS Statistics v28 (IBM Corp., Armonk, NY). Fecal cortisol metabolite concentrations were analyzed using a mixed-design repeated-measures general linear model. Transition stage was specified as a four-level within-cow factor, corresponding to 1 month before parturition (1 mbp), 1 week before parturition (1 wbp), 1 week after parturition (1 wap), and 1 month after parturition (1 map). Season (winter vs. summer) and farm were included as between-subject factors. Because different cows were sampled in winter and summer, Season represented a between-cow rather than a within-cow comparison. The individual cow was the subject and experimental unit for longitudinal inference.

The model evaluated the main effects of transition stage, Season, and Farm, together with the Stage × Season, Stage × Farm, Season × Farm, and Stage × Season × Farm interactions. Farm was included to account for systematic between-herd heterogeneity under commercial conditions. Because only four farms were represented and farm-level management and structural characteristics were not independently replicated, farm-related effects were not interpreted causally or used to infer effects of specific management practices.

Sphericity of the repeated-measures factor was assessed using Mauchly’s test. The sphericity assumption was not rejected (W = 0.777, χ^2^[5] = 7.247, *P* = 0.203); therefore, uncorrected degrees of freedom were used for the primary within-subject tests. Greenhouse–Geisser-corrected results were also available from the model and led to the same inference for the main effect of Stage.

Estimated marginal means (EMMs), standard errors, and 95% confidence intervals were obtained for each transition stage. Pairwise comparisons among stages were adjusted using the Bonferroni procedure. Because interpretation of season-specific stage contrasts depends on evidence of effect modification by Season, the Stage × Season interaction was evaluated before interpreting simple effects.

Effect sizes for omnibus tests were expressed as partial eta squared (η^2^p), calculated from the corresponding Type III sums of squares. Statistical tests were two-sided, and *P* < 0.05 was considered statistically significant. Exact *P*-values are reported where available.

Residual distributions and variance structure were assessed by visual inspection of Q-Q and residual-versus-fitted plots. Homogeneity of variance across Farm × Season groups was evaluated at each transition stage using mean-based Levene and median-based Brown–Forsythe tests. Robustness of the main Stage effect was further assessed after log transformation of FCM concentrations, by leave-one-cow-out analyses, and by refitting the repeated-measures model without Farm.

### Data availability statement

The verified dataset supporting the revised analyses is available from the corresponding author upon reasonable request.

## Results

### Longitudinal FCM dynamics across the transition period

Fecal cortisol metabolite concentrations varied significantly across transition stages (F[3, 90] = 13.17, *P* < 0.001, partial eta squared = 0.305). In contrast, the overall effect of Season was not significant (F[1, 30] = 0.056, *P* = 0.815), and the Stage × Season interaction, averaged across farms, was not significant (F[3, 90] = 1.53, *P* = 0.213). The effects of transition stage, season, farm, and their interactions on FCM concentrations are summarized in Table 2.

**Table 2:** Effects of transition stage, season, farm, and their interactions on fecal cortisol metabolite concentrations in the repeated-measures analysis.

Factor	Numerator df	Denominator df	F-value	*P*-value	Partial eta squared
**Stage**	3	90	13.167	<0.001	0.305
**Season**	1	30	0.056	0.815	0.002
**Farm**	3	30	27.878	<0.001	0.736
**Stage × Season**	3	90	1.527	0.213	0.048
**Stage × Farm**	9	90	3.637	<0.001	0.267
**Season × Farm**	3	30	6.293	0.002	0.386
**Stage × Season × Farm**	9	90	2.089	0.039	0.173

Abbreviations: df, degrees of freedom; FCM, fecal cortisol metabolites. Stage was the within-cow factor; Season and Farm were between-subject factors. The analysis included 38 cows with four repeated measurements each. Within-subject tests use the sphericity-assumed degrees of freedom. Partial eta squared was calculated as the Type III sum of squares for the effect divided by the sum of that effect and its corresponding error sum of squares. Farm-related effects describe heterogeneity among the sampled herds and do not establish causal effects of specific farm characteristics or management practices.

Estimated marginal means showed an overall decline from the prepartum to the postpartum period. Mean FCM concentrations were 421.1 ng/g dry feces (SE = 28.2; 95% CI, 363.5–478.8) at 1 month before parturition, 342.2 ng/g (SE = 26.2; 95% CI, 288.8–395.6) at 1 week before parturition, 238.5 ng/g (SE = 16.1; 95% CI, 205.6–271.5) at 1 week after parturition, and 226.1 ng/g (SE = 26.9; 95% CI, 171.1–281.0) at 1 month after parturition.

Bonferroni-adjusted pairwise comparisons showed that FCM concentrations were higher at 1 month before parturition than at 1 week after parturition (mean difference = 182.6 ng/g; 95% CI, 84.8–280.4; *P* < 0.001) and 1 month after parturition (mean difference = 195.1 ng/g; 95% CI, 92.0–298.2; *P* < 0.001). Concentrations at 1 week before parturition were also higher than at 1 week after parturition (mean difference = 103.7 ng/g; 95% CI, 16.0–191.3; *P* = 0.013). The remaining comparisons were not statistically significant: 1 month before versus 1 week before parturition (*P* = 0.365), 1 week before versus 1 month after parturition (*P* = 0.053), and 1 week after versus 1 month after parturition (*P* = 1.000). All six pairwise comparisons, including mean differences, standard errors, Bonferroni-adjusted confidence intervals, and P-values, are presented in Table S3.

Diagnostic evaluation indicated some departure from normality and variance homogeneity on the raw FCM scale, most clearly at 1 month after parturition (Fig. S1). Median-based Brown–Forsythe tests did not reject homogeneity of variance at any stage (all *P* ≥ 0.089), whereas mean-based Levene tests indicated unequal variance at 1 week before parturition (*P* = 0.032) and 1 month after parturition (*P* = 0.013). Log transformation improved the residual distribution and variance homogeneity, with non-significant mean-based Levene tests at all four stages (all *P* ≥ 0.211; Fig. S2). The Stage effect remained significant after log transformation (F[3, 90] = 20.17, *P* < 0.001) and in all leave-one-cow-out analyses (F range, 11.74–18.54; all *P* < 0.001). High FCM observations were retained because there was no documented basis for excluding them as erroneous. In an additional sensitivity analysis without Farm, Stage remained significant (F[3, 108] = 10.51, *P* < 0.001), whereas Season (F[1, 36] = 0.049, *P* = 0.826) and the Stage × Season interaction (F[3, 108] = 1.33, *P* = 0.269) remained non-significant.

### Descriptive commercial-farm context and between-farm variability

FCM concentrations differed among the four commercial farms included in the study (Farm effect: F[3, 30] = 27.88, *P* < 0.001, partial eta squared = 0.736). A significant Season × Farm interaction was also observed (F[3, 30] = 6.29, *P* = 0.002, partial eta squared = 0.386). The longitudinal FCM profile also varied among farms, as indicated by the Stage × Farm interaction (F[9, 90] = 3.64, *P* < 0.001, partial eta squared = 0.267) and the Stage × Season × Farm interaction (F[9, 90] = 2.09, *P* = 0.039, partial eta squared = 0.173).

These results indicate heterogeneity among the four sampled herds. However, because only four farms were included and farm-level characteristics were not independently replicated, these effects were not interpreted as evidence of causal effects of particular housing, production-system, nutritional, or management characteristics. Farm-specific FCM values are therefore presented in Tables S1 and S2 to document the variability observed under the commercial conditions represented in the study.

## Discussion

This longitudinal field study identified significant variation in fecal cortisol metabolite concentrations across transition stages in 38 multiparous dairy cows, based on 152 repeated observations. Concentrations showed an overall prepartum-to-postpartum decline. However, significant Stage × Farm and Stage × Season × Farm interactions indicated that the longitudinal profile varied among the sampled herds, requiring caution when generalizing the overall pattern across farms and seasons. Higher prepartum FCM concentrations are consistent with greater adrenocortical activity during this period. This interpretation is biologically plausible given the endocrine, metabolic, immune, and behavioral adjustments surrounding parturition and the onset of lactation (Abuelo et al. 2014, 2016; Sundrum 2015; Mezzetti et al. 2020).

However, FCM concentrations alone cannot determine whether the observed pattern represents effective adaptation, maladaptation, distress, or impaired welfare. Accordingly, the present findings should be interpreted as a longitudinal change in FCM concentrations consistent with variation in adrenocortical activity, rather than as direct evidence of the biological or welfare consequences of that response. Because FCM reflect glucocorticoid secretion occurring several hours before fecal excretion, the standardized 09:00–11:00 h collection window helped reduce sampling-time variability but does not identify the precise timing of the preceding endocrine response. However, a fixed fecal sampling window does not establish that circadian influences were fully controlled. Sampling time relative to feeding and milking was not included in the analysis, so potential variation associated with these routines could not be separated from the observed FCM pattern.

The present findings extend previous experimental and cross-sectional studies by showing that this longitudinal pattern was detectable in the commercial herds included in the study. Repeated, low-disturbance FCM profiling captured a consistent overall prepartum-to-postpartum pattern without disrupting routine herd management.

From an applied animal science perspective, repeated FCM measurements may provide a complementary method for characterizing periods of increased physiological demand under commercial conditions. The present study demonstrates the feasibility of longitudinal FCM profiling but does not establish diagnostic thresholds, predictive performance, individual repeatability, or clinical decision limits. FCM should therefore be interpreted alongside clinical, productive, behavioral, environmental, and welfare indicators rather than as a stand-alone monitoring tool.

The use of fecal cortisol measurements is particularly relevant under field conditions because low-disturbance fecal sampling provides a less invasive alternative to repeated blood sampling and reflects medium-term adrenocortical activity, reducing the influence of acute stress and pulsatile cortisol release (Möstl et al. 2002; Bertulat et al. 2013; Ebinghaus et al. 2020; Idris et al. 2024).

Stress responses in dairy cattle are multifactorial and may be influenced by nutritional status, energy balance, environmental conditions, housing, stocking density, and human-animal interactions (Jacob et al. 2001; Wathes et al. 2007; Huzzey et al. 2012; Bova et al. 2014; Jurkovich et al. 2024). These factors were not independently tested in the present study and should be considered possible contributors to individual and between-farm variability rather than explanatory variables.

The overall Season effect and the Stage × Season interaction were not statistically significant. These results provide no evidence of an overall seasonal difference in FCM concentrations or of a Stage × Season interaction averaged across farms; they do not establish equivalence between seasons. Moreover, the significant Season × Farm and Stage × Season × Farm interactions indicate that seasonal patterns differed among farms and that the Stage × Season relationship was not uniform across herds. These findings should therefore be interpreted within the specific commercial settings represented in this study. Temperature-humidity index (THI) values were used solely to describe the environmental context and were not included as an inferential covariate in the repeated-measures analysis. Accordingly, this analysis does not establish an association between THI and FCM concentrations or support attribution of the observed FCM changes to heat stress. Because THI was summarized at the farm-season level, short-term thermal exposure, maximum THI values, and the duration of exposure above specific heat-load thresholds were not characterized.

Farm-level information was included to document the commercial conditions under which FCM measurements were obtained. These descriptors cannot be interpreted as causal factors because they were not independently replicated and are confounded with farm-specific effects. The observed variability reinforces the importance of evaluating FCM responses within a longitudinal, within-animal framework.

The study was based on 38 cows from four commercial farms, with four repeated measurements per cow, yielding 152 longitudinal observations. These repeated observations characterize within-cow changes but do not represent independent animals; the cow remains the unit for longitudinal inference. The limited number of cows and farms constrains the precision and generalizability of the findings, particularly for interactions involving farm and season. Furthermore, farm-level management and structural characteristics were not independently replicated, precluding causal inference regarding specific farm descriptors. Although the repeated-measures design supports assessment of changes across transition stages, the observed heterogeneity among farms requires caution when extrapolating the overall pattern to other herds or management settings. The analysis was restricted to available complete longitudinal records, and potential selection bias associated with record availability cannot be excluded.

Additional limitations concern factors that could contribute to the observed FCM pattern but were not accounted for in the present analysis. Although feeding regimens were described, individual feed intake and energy balance were not measured, and the analysis did not separate potential dietary or metabolic influences from changes associated with transition stage. Clinical screening at selection does not exclude subclinical disease or health problems developing during follow-up. Potential contributions from dystocia, twinning, retained placenta, metritis, and lameness were not evaluated in the FCM analysis. These factors could influence both individual concentrations and differences among farms or stages. Consequently, the observed prepartum-to-postpartum decline cannot be attributed exclusively to physiological stage or interpreted as evidence of effective adaptation or impaired welfare. Future studies should integrate repeated FCM measurements with individual nutritional, metabolic, clinical, reproductive, productive, and behavioral records to evaluate these potential sources of variation. Although cows were selected as multiparous and within a body condition score range of 3.0–3.5, individual parity number, variation in body condition, and milk yield were not included in the FCM analysis. Their potential contributions to the observed differences therefore remain unresolved.

The higher prepartum FCM concentrations identify this phase as a relevant window for investigating physiological demand in commercial dairy cows. However, additional studies are needed to determine individual repeatability, establish reference intervals and clinically relevant thresholds, and assess whether FCM measurements are associated with subsequent health, production, behavioral, or welfare outcomes. In addition, because cows with evidence of clinical disease were excluded during animal selection, the observed FCM range may have been narrower than that occurring across the full spectrum of animals in commercial herds. The present findings therefore primarily characterize longitudinal FCM patterns in clinically healthy multiparous cows and should not be extrapolated to the full range of stress responses associated with clinical disease.

## Conclusions

In this longitudinal observational study of 38 clinically healthy multiparous dairy cows with 152 repeated observations, FCM concentrations varied across transition stages, showing an overall prepartum-to-postpartum decline. Neither the overall Season effect nor the Stage × Season interaction was significant, although farm-related interactions indicated variation among the sampled herds. The overall pattern was characterized by higher FCM concentrations at prepartum than postpartum stages, although not all pairwise comparisons were statistically significant. These observations do not establish the physiological causes of the pattern, adverse health or welfare consequences, or causal effects of specific management or environmental conditions. Further studies integrating endocrine measurements with clinical, productive, behavioral, and environmental indicators are needed to establish its practical relevance.

## Supplementary Material

txag174_Supplementary_Data

## Data Availability

The verified dataset supporting the revised analyses is available from the corresponding author upon reasonable request.

## CRediT author statement

C. Castillo: Conceptualization, Formal analysis, Investigation, Writing—original draft, Writing—review and editing. L. Díaz: Investigation, Methodology, Writing—review and editing. E.N. Martínez: Formal analysis, Methodology, Visualization, Writing—review and editing. R. Muiño: Funding acquisition, Resources, Writing—review and editing. J. Hernández: Data curation, Formal analysis, Software, Writing—review and editing.

## List of abbreviations

AP: after parturition
BP: before parturition
EIA: enzyme immunoassay
FCM: fecal cortisol metabolites
HPA: hypothalamic-pituitary-adrenal
THI: temperature-humidity index
TMR: total mixed ration
WIN: winter
SUM: summer
mbp: month before parturition
wbp: week before parturition
wap: week after parturition
map: month after parturition
