# Supplementary material for: Longitudinal fecal cortisol metabolite concentrations across the transition period in multiparous dairy cows under commercial conditions
Source: Transl Anim Sci. 2026 Oct 28;10:txag174. doi: 10.1093/tas/txag174 (PMC13769239; doi:10.1093/tas/txag174)
Supplement: Supplementary file 1 [file txag174_supplementary_data.docx]

Supplementary Table S1. Descriptive mean values (± standard error) of fecal cortisol metabolite concentrations (ng/g dry feces) by farm, transition stage, and season

Values are presented only to document the range of FCM concentrations observed across the four commercial dairy farms. They should not be interpreted as tests of production-system, structural, or management effects because farm-level descriptors were not independently replicated.

| **Season** | **Stage** | **Farm 1 (Intensive)** | **Farm 2 (Intensive)** | **Farm 3 (Semi-intensive)** | **Farm 4 (Intensive)** |
| --- | --- | --- | --- | --- | --- |
| WIN | 1 mbp | 277.4 ± 35 | 395.9 ± 158 | 324.1 ± 15 | 721.2 ± 87 |
|  | 1 wbp | 430.2 ± 66 | 205.8 ± 116 | 251.3 ± 26 | 604.8 ± 95 |
|  | 1 wap | 216.3 ± 18 | 183.7 ± 48 | 294.0 ± 79 | 234.3 ± 20 |
|  | 1 map | 170.0 ± 28 | 190.0 ± 28 | 153.3 ± 14 | 215.8 ± 37 |
| SUM | 1 mbp | 595.4 ± 112 | 232.4 ± 25 | 254.2 ± 36 | 568.5 ± 83 |
|  | 1 wbp | 327.4 ± 57 | 131.0 ± 30 | 140.6 ± 23 | 646.3 ± 110 |
|  | 1 wap | 256.2 ± 43 | 246.9 ± 54 | 189.4 ± 37 | 287.3 ± 38 |
|  | 1 map | 549.1 ± 157 | 60.0 ± 15 | 93.2 ± 13 | 377.2 ± 114 |

Abbreviations: WIN, winter; SUM, summer; 1 mbp, 1 month before parturition; 1 wbp, 1 week before parturition; 1 wap, 1 week after parturition; 1 map, 1 month after parturition. Farm-level values are descriptive only and should not be interpreted as tests of causal farm-level effects.

The dataset comprised 38 cows from four commercial farms (Farm 1, n = 10; Farm 2, n = 8; Farm 3, n = 10; Farm 4, n = 10), with 19 cows sampled in winter and 19 different cows sampled in summer. Each cow contributed four repeated measurements, yielding 152 longitudinal observations. Values are descriptive means ± standard errors for each farm, season, and stage.

Supplementary Table S2. Descriptive mean values (± standard error) of fecal cortisol metabolite concentrations (ng/g dry feces) in the intensive-farm subset, by farm, transition stage, and season

| **Season** | **Stage** | **Farm 1** | **Farm 2** | **Farm 4** |
| --- | --- | --- | --- | --- |
| WIN | 1 mbp | 277.4 ± 35 | 395.9 ± 158 | 721.2 ± 87 |
|  | 1 wbp | 430.2 ± 66 | 205.8 ± 116 | 604.8 ± 95 |
|  | 1 wap | 216.3 ± 18 | 183.7 ± 48 | 234.3 ± 20 |
|  | 1 map | 170.0 ± 28 | 190.0 ± 28 | 215.8 ± 37 |
| SUM | 1 mbp | 595.4 ± 112 | 232.4 ± 25 | 568.5 ± 83 |
|  | 1 wbp | 327.4 ± 57 | 131.0 ± 30 | 646.3 ± 110 |
|  | 1 wap | 256.2 ± 43 | 246.9 ± 54 | 287.3 ± 38 |
|  | 1 map | 549.1 ± 157 | 60.0 ± 15 | 377.2 ± 114 |

No causal interpretation should be made for structural or management descriptors because individual farm characteristics were not independently replicated.

Abbreviations: WIN, winter; SUM, summer; 1 mbp, 1 month before parturition; 1 wbp, 1 week before parturition; 1 wap, 1 week after parturition; 1 map, 1 month after parturition. Values are descriptive only and document within-subset farm-level variability; they should not be interpreted as tests of structural or management effects.

Supplementary Table S3. Bonferroni-adjusted pairwise comparisons of estimated marginal mean fecal cortisol metabolite concentrations across transition stages.

| Comparison | Mean difference (ng/g dry feces) | SE | Adjusted 95% CI | Adjusted P-value |
| --- | --- | --- | --- | --- |
| 1 mbp − 1 wbp | 78.952 | 40.541 | −35.563 to 193.467 | 0.365 |
| 1 mbp − 1 wap | 182.622 | 34.615 | 84.844 to 280.399 | <0.001 |
| 1 mbp − 1 map | 195.069 | 36.495 | 91.981 to 298.156 | <0.001 |
| 1 wbp − 1 wap | 103.670 | 31.024 | 16.036 to 191.304 | 0.013 |
| 1 wbp − 1 map | 116.117 | 41.504 | −1.120 to 233.353 | 0.053 |
| 1 wap − 1 map | 12.447 | 29.829 | −71.811 to 96.705 | 1.000 |

Comparisons are based on estimated marginal means averaged across Season and Farm. Differences represent the first stage minus the second stage. Confidence intervals and P-values are adjusted using the Bonferroni procedure for the six pairwise comparisons. P-values below 0.001 are reported as displayed in the original SPSS output.

Abbreviations: SE, standard error; CI, confidence interval; 1 mbp, 1 month before parturition; 1 wbp, 1 week before parturition; 1 wap, 1 week after parturition; 1 map, 1 month after parturition.

Supplementary Figure S1. Diagnostic plots for the repeated-measures analysis of raw fecal cortisol metabolite concentrations. Q-Q plots and residual-versus-fitted plots are shown for each transition stage to assess residual distribution and variance structure.

Supplementary Figure S2. Diagnostic plots for the repeated-measures analysis after logarithmic transformation of fecal cortisol metabolite concentrations. Q-Q plots and residual-versus-fitted plots are shown for each transition stage. The logarithmic transformation improved residual distribution and variance homogeneity relative to the raw scale.
